# Editorial: Plant-Arthropod Interactions: Effectors and Elicitors of Arthropods and Their Associated Microbes

**DOI:** 10.3389/fpls.2020.610160

**Published:** 2020-11-04

**Authors:** Akiko Sugio, Gary W. Felton, David Giron, Isgouhi Kaloshian

**Affiliations:** ^1^INRAE, UMR1349, Institute of Genetics, Environment and Plant Protection, Le Rheu, France; ^2^Department of Entomology, Pennsylvania State University, University Park, PA, United States; ^3^Centre National de la Recherche Scientifique, Tours, France; ^4^Department of Nematology, Institute for Integrative Genome Biology, University of California, Riverside, Riverside, CA, United States

**Keywords:** plant, insect, arthropod, herbivore, elicitor, effector

With the advent of omics technologies, sequencing of genomes and transcriptomes of a number of arthropods have been accomplished. These achievements have brought about a renaissance in the study of host plant and herbivorous arthropod interactions. Using these approaches, intricate interactions have been revealed. Secretions from arthropods presumably delivered into the host plant and containing proteinaceous effectors and elicitors of both arthropod and microbial origins, were shown to modulate plant immunity and metabolism acting as inducers or suppressors of physiological responses. In this Research Topic, we aimed to gather research articles and reviews that describe the identification and characterization of the effectors and the elicitors involved in the interactions with host plants.

For many herbivores, the first contact with their host plant is during egg deposition or oviposition. Plants have evolved sensing mechanisms to recognize the mechanical and chemical cues associated with oviposition. In their review, Bertea et al. provide an excellent overview of the responses of host plants to oviposition by Lepidoptera (i.e., moths and butterflies). Egg-induced defenses can directly impair or kill eggs through localized necrosis, neoplasm formation, and/or the direct production of ovicidal compounds. Plants also produce oviposition-induced plant volatiles which attract parasitoids that eventually kill the eggs or larvae. They argue that progress in understanding the specificity of these responses requires further characterization of egg-associated elicitors and the plant receptors that recognize these chemical cues. Gouhier-Darimont et al. contribute an important paper in understanding plant perception of oviposition. In Arabidopsis, eggs of the specialist butterfly, *Pieris brassicae* elicit a burst of reactive oxygen species and salicylic acid, and downstream defense gene expression and localized necrosis. Oviposition and egg cues trigger the localized expression of an L-type lectin receptor kinase LecRK-I.8. Using an Arabidopsis knock-out mutant *lecrk-I.8*, they found that the plant defense responses to these egg cues were significantly impaired in this mutant. Their results demonstrate that LecRK-I.8 is an early component of egg perception.

After the eggs hatch, herbivorous arthropods start to feed on their host plants and direct interactions between the animal and the plant begin. Tomato responses to two spider mite species, *Tetranychus urticae* and *T. evansi* were examined in detail by analyzing expression patterns of marker genes for jasmonic and salicylic acids defense hormones (Liu et al.). In this analysis, they compared cumulative effect of mite life stages and effect of feeding by male and female adult mites. They also examined salivary effector expression patterns in similar cohorts of mites. Their study shows complex interactions of spider mites and their host and demonstrates fine-tuned regulation of salivary effector expressions in the two mite species.

While feeding, herbivorous arthropods secrete proteinaceous saliva. Various salivary components are identified and analyzed. Liu and Bonning took advantage of the enhanced availability of genomic resources for stink bugs to explore the repertoire of digestive enzymes through a tissue-specific transcriptome analysis. Their work provides evidence for the principal salivary gland being the primary source of proteases and nucleases used for efficient digestion of plant materials. They also show that *Halyomorpha halys* and *Nezara viridula* have a similar digestive biochemical arsenal and propose that the large diversity of salivary enzymes may mediate the ability of stink bugs to feed on multiple hosts. The ability of stink bugs to feed on diverse crop systems is further explored by CantÓn and Bonning. They demonstrate that protease and nuclease activity of *N. viridula* maintained on different plant diets are similar. Conversely, their work shows that specific transcripts of the digestive enzymes are different. How diet could change the digestive physiology may help understand polyphagy and could open new avenues for the development of innovative control strategies of pests. Nevertheless, the study is limited in its finding because of inadequate genomic resources.

A thorough comparative analysis of salivary gene expression patterns in *Acyrthosiphon pisum* biotypes, which show distinct host plant specificity, reveal that the majority of the genes encoding candidate salivary effectors are expressed in two biotypes compared, and that there are small subsets of genes that are differentially expressed in a biotype-specific manner (Boulain et al.). As those subsets are enriched with duplicated and aphid-lineage-specific genes, the authors propose a scenario that biotype-specific salivary effectors have evolved recently and diversified through duplication events. Further, two candidate salivary effector families are reported in *A. pisum* (Dommel et al.). The members of these gene families encode highly conserved secretory signal peptides and divergent mature proteins derived from miniature exons. The family members are scattered throughout *A. pisum* genome and encoded in unusually large genomic regions. The authors propose a model that the gene families expanded in *A. pisum* through combinatorial assemblies of a common secretory signal cassette and novel coding regions, and hypothesis that the gene families facilitate the adaptation of the aphid to new hosts. MacWilliams et al. profile the salivary proteome of the cowpea aphid, *Aphis craccivora*. Their work identifies a novel effector, AcDCXR, a member of short-chain dehydrogenases/reductases. They show that the recombinant AcDCXR protein has the predicted enzymatic activity in carbohydrate and dicarbonyl metabolisms with putative ability to enhance nutrition to the aphid as well as alter plant defense responses. Consistently, they show that transient expression of AcDCXR enhances the fecundity of the aphid. Their work also provides evidence for the existence of a novel pest defense metabolite, methylglyoxal, known for its role in abiotic stress.

Effectors are recognized by plant resistance (R) proteins and a way to overcome this resistance is the ability of the pest to mutate the effector to evade the recognition by the cognate R protein. Navarro-Escalante et al. describe the use of bulked-segregant analysis and whole genome sequence to identify virulent effectors from the Hessian flies (*Mayetiola destructor*) that have overcome single gene resistances in wheat. Their work confirms the identity of a previously identified virulence effector *vH6*, as well as identifies a second virulence effector *vHdic*. Using heterologous expression system, they show the ability of these two virulence effectors to suppress plant immune responses providing direct evidence for the role of effectors in pest virulence.

Taken together, these articles demonstrate that new technologies clearly expanded the opportunities to study a wide range of arthropod-plant interactions. These resources enabled identification of numerous effector/elicitor candidates, description of their expression patterns and their receptors. Yet, functional characterization of effectors/elicitors remains a big challenge. Model systems (e.g., Arabidopsis) could advance the field rapidly, but arthropod host specificity limits the use of model systems. In some cases, heterologous systems can be employed to overcome these difficulties. Further development of research tools is needed to understand functions of effectors, perception mechanisms of elicitors and how these activities are translated into the interactions between plants and herbivores.

## Author Contributions

All authors listed have made a substantial, direct and intellectual contribution to the work, and approved it for publication.

## Conflict of Interest

The authors declare that the research was conducted in the absence of any commercial or financial relationships that could be construed as a potential conflict of interest.

